# Attention Bias and Anxiety: The Moderating Effect of Sociocultural Variables in Rural Latinx Youth

**DOI:** 10.1007/s10597-023-01132-y

**Published:** 2023-05-06

**Authors:** Elizabeth S. Bocanegra, Susanna W. Chang, Michelle Rozenman, Steve S. Lee, Desiree Delgadillo, Denise A. Chavira

**Affiliations:** 1 Department of Psychology, University of California Los Angeles, 502 Portola Plaza, Los Angeles, CA 90095 USA; 2 Division of Child and Adolescent Psychiatry, Semel Institute for Neuroscience and Human Behavior, University of California, Los Angeles, CA USA; 3 Department of Psychology, University of Denver, Denver, CO USA

**Keywords:** Attention bias, Latinx, Youth, Anxiety, Poverty

## Abstract

Attention bias confers risk for anxiety development, however, the influence of sociodemographic variables on the relationship between attention bias and anxiety remains unclear. We examined the association between attention bias and anxiety among rural Latinx youth and investigated potential moderators of this relationship. Clinical symptoms, demographic characteristics, and a performance-based measure of attention bias were collected from 66 rural Latinx youth with clinical levels of anxiety (33.3% female; *M*_age_ = 11.74; 92.4% Latinx, 7.6% Mixed Latinx). No moderating effects for age or gender were found. Youth below the poverty line displayed an attention bias *away* from threat in comparison to youth above the poverty line, who displayed an attention bias *towards* threat. Among youth below the poverty line, this bias away from threat was associated with increased anxiety. Findings highlight the importance of economic adversity in understanding the relationship between attention bias and anxiety.

## Introduction

Attention is a key feature in the encoding stage of cognitive processing. With countless inputs being perceived at a given moment, prioritizing certain kinds of stimuli in preference of others is essential for effective information processing. While attending to threats in the environment efficiently streamlines and facilitates information processing, biases can arise during this stage of processing. Attention bias can be defined as a tendency to overattend to emotionally threatening stimuli over neutral stimuli, which can lead to an overgeneralization that one’s surroundings are unsafe thereby increasing the frequency, duration, and intensity of fear responses (Azriel & Bar-Haim, 2020). Clinical and experimental theories suggest that processing biases in attention potentiate cognitive representation of events in a way that directly mediates vulnerability to anxiety (Beck & Clark, 1997; Williams et al., 1988). Cognitive models of anxiety further assert that anxious individuals preferentially attend to threatening information in their environment during the initial stages of cognitive processing, which downstream impacts higher levels of cognition, including negative interpretation bias, encoding, and subsequent memory of such information, ultimately leading to anxious behaviors such as avoidance and reassurance-seeking.

Some studies have found that attention bias is central to the etiology and maintenance of psychological disorders (Bar‐Haim, 2010), including evidence that the inability to disengage attention from threat-related probes in emotionally relevant information (Derakshan & Eysenck, 2009) elevates risk of internalizing disorders (Bar-Haim et al., 2007; MacLeod et al., 1986; Mogg et al., 1995). The evaluation of emotional valence at the beginning stages of cognitive processing, a process that is usually automatic and without conscious awareness (LeDoux, 1995, 1996; Öhman, 1993), often results in a preference towards threat-detection in anxious individuals, creating hypervigilance for subsequent threat (Bar-Haim et al., 2007). Anxiety symptoms have been linked with this sustained and more automatic attention for threatening stimuli (Mogg & Bradley, 2005), perpetuating a cycle of excessively identifying and negatively interpreting potential threats from the environment.

Studies report that attentional biases begin early in development. In youth, attention bias towards threat has been shown to be positively associated with poor emotional regulation and overgeneralization of fear learning (Shechner et al., 2013, 2015). The presence of attentional bias towards threat over the course of a child’s development has been shown to be associated with an increased risk of developing an anxiety disorder (Muris & Ollendick, 2005; Nigg, 2006). While a positive association between attention bias and anxiety has been found in many studies, (Pineles & Mineka, 2005; Pishyar et al., 2004; Waters et al., 2014), other studies have not found a significant association (Heeren et al., 2015). At present, many studies rely on cross-sectional designs, and sociodemographic variables have been largely ignored when considering this association. Further, when sociodemographic variables have been considered, samples have included primarily non-Latinx White individuals (Shechner et al., 2013).

Experimental studies that utilize computer-based interventions to target attention bias (i.e., Attention Bias Modification training; ABM), given its hypothesized role as a mechanism of change for anxiety reduction, have found that such interventions are efficacious, though findings have been mixed. A number of randomized controlled trials have demonstrated that ABM protocols focused on training attention away from threat result in notable anxiety symptom reduction relative to a control condition (Hakamata et al., 2010; Linetzky et al., 2015; Price et al., 2016). Moreover, in many studies, albeit not all, the correlation between change in attention bias and improvement in anxiety symptoms has been significant (Britton et al., 2013; Eldar et al., 2012; Gotlib et al., 2004). At the same time, other treatment studies have not found significant differences in anxiety symptom reduction among those who received an ABM intervention compared to those in the control condition. These latter findings are often explained by nonsignificant changes in attention bias among participants who receive, the possibility of third variables that lead to change in both conditions (e.g., attentional control) (Ollendick et al., 2019; Pergamin‐Hight et al., 2016; Pettit et al., 2020), and methodological factors, such as small, heterogeneous samples, use of restricted age ranges, and different testing contexts (e.g., laboratory settings, scanner settings, participants’ homes) (Roy et al., 2008).

Recently, a review from Fu and Pérez-Edgar (2019) suggested that the inconsistencies found in youth attention bias research could be due to considerable individual differences influencing the relationship between attention bias and anxiety in this group. Indeed, conceptual frameworks of attention bias development (Morales et al., 2016) posit that individual differences in children, coupled with experiences in their environment, modulate attention bias development and whether it subsequently becomes maladaptive. Moderators may therefore explain some of the variability in findings across studies examining attention bias and anxiety (MacKinnon, 2011). Broader literature has indicated that cognitive processes such as attentional bias may vary across age, gender, race/ethnicity and socioeconomic groups, in part due to the unique stressors, environments, and experiences that individuals from within these groups encounter (Williams & Mohammed, 2009). To our knowledge, the moderating influence of specific sociodemographic factors on the relationship between attention bias and anxiety has been limited.

Data suggest there are shifts in attention bias across age and development, and one study has found that age is a moderator of attention bias in youth (Carmona et al., 2015). Prior to adolescence, youth show an attentional preference towards both negative and positive emotional stimuli, however over time, there is a shift towards a preference for negative stimuli (Burris et al., 2017; Elam et al., 2010; Grose-Fifer et al., 2009). This shift is likely influenced by increased social pressure and intense emotional experiences common among teenagers (Casey et al., 2010; Zhao et al., 2014) as well as developmental shifts in maturing neurological pathways, which increase vulnerability for social anxiety and other anxiety disorders in adulthood (Brozovich & Heimberg, 2008; Mychailyszyn et al., 2010; Woodward & Fergusson, 2001). Once problems such as social anxiety emerge, teens may have further difficulty disengaging their attention from potentially threatening social cues, a consequence and maintaining factor for attention bias (Cisler & Olatunji, 2010; Moriya & Tanno, 2011; Yiend & Mathews, 2001). Although data are limited, studies suggest that age may have a moderating influence on the association between attention bias and anxiety.

Data also reveal differential endorsement of anxious symptoms between girls and boys in childhood and adolescence, with girls reporting more anxiety symptoms than boys (Costello et al., 2003; Merikangas et al., 2010). Some studies suggest that girls have a greater cognitive bias toward negative stimuli than boys (Miers et al., 2008; Salemink & Wiers, 2011), a bias which may partially account for adolescent girls reporting greater and more severe anxiety symptoms (Costello et al., 2003; Merikangas et al., 2010). These findings, while limited, suggest that gender also may be an important variable to consider when examining the impact of cognitive processes on anxiety (Sherman & Ehrenreich-May, 2018).

Lastly, youth who experience socioeconomic disadvantage are more likely to develop anxiety compared to those from middle or high socioeconomic groups (Lemstra, 2008). A wide variety of mechanisms have been implicated in the relationship between poverty and internalizing disorders, including influences at the individual, family, and neighborhood level (Bradley & Corwyn, 2002). For example, poverty is associated with increased exposure to adversity, including harsh parenting (Repetti et al., 2002) and exposure to violence (Sampson et al., 1997), which have been found to increase risk for internalizing disorders (Hurt et al., 2001; Kingsbury et al., 2020). Specific to Latinx youth, studies have found that toxin exposure in youths' physical environments disrupts the automatic nervous system and the body’s stress response system (Ugarte et al., 2022), thereby creating a pathway to increased susceptibility to anxious and depressive symptoms.

Indeed, economic disadvantage and adverse life experiences (including trauma exposure) may lead to attentional biases (Bradley & Corwyn, 2002; Loomis, 2021), which may confer risk for internalizing disorders. In an fMRI study with youth ages 9–18 years old, early-life family adversity was associated with neural overactivation when processing threatening stimuli (Maheu et al., 2010). In a structural MRI study of youth between the ages of 8–10, findings supported income related variations in brain structure and attention bias to threat (Dufford et al., 2019)—the role of socioeconomic status as a moderator of attention bias and anxiety symptoms was not examined. Additionally, childhood material deprivation has been linked to greater physiological reactivity to ambiguous social situations, as shown by increased diastolic blood pressure and heart rate reactivity (Chen et al., 2004)—the negative interpretation of ambiguous situations may be particularly relevant for biases arising in attention processes. Cumulatively these findings suggest that poverty and early adversity influence attention bias to threat and may be risk factors for the development of psychopathology.

Overall, the relationship between attention bias and anxiety may shift with variations in population, context, and current affective state (Morales et al., 2015). Latinx youth are a rapidly growing subset of the U.S. population (Fry & Gonzales, 2008) and report higher rates of anxiety symptoms and disorders than White and Black youth (Knopf et al., 2008; MacKay, 2008). Youth from rural Latinx communities may be at heightened risk for mental health problems, due to well-documented challenges in these areas, including lack of access to health services (Raffaelli & Wiley, 2013; Taylor & Ruiz, 2017), elevated rates of loneliness (Stacciarini et al., 2015), poverty (Raffaelli & Wiley, 2013), and high levels of discrimination (Finch et al., 2000). Given the multiplicity of economic and sociodemographic stressors as well as elevated rates of anxiety (Fontanella et al., 2015; Polaha et al., 2011), rural Latinx youth represent a particularly important population to consider when examining potential risk factors for anxiety such as attention bias and moderating influences.

The aim of this study was (1) to understand the relationship between attention bias and anxiety in a group of socio-economically diverse rural Latinx youth, and (2) to examine moderators of the relationship between attention bias and anxiety, such as age, gender and poverty. Based on existing literature, we hypothesized that attention bias would be significantly related to anxiety in this sample and that being female, as well as being older, would be associated with increased attention bias, and higher levels of anxious symptomology. Analyses also were conducted to examine whether poverty moderated the relationship between attention bias and anxiety. To date, there have not been any studies focusing on the relationship between attention bias and anxiety among Latinx or rural youth, an at-risk sample, for whom the effects of anxiety are particularly pronounced.

## Methods

### Recruitment

Data were collected as part of a larger randomized controlled trial (RCT) for youth with anxiety symptoms from a rural Latinx community in southern California. As of the 2006 Census, approximately 75% of the population in this county were of Latinx descent (predominantly Mexican), relative to 30.6% in the State of California. The majority of individuals in this county speak Spanish as their first language (65%). Many households (46.7%) have children under the age of 18, and 22.6% of the population is below the poverty line, making it the most impoverished county in California. Families were recruited from 10 community health care clinics that are part of the local healthcare system. This healthcare system also has a trained community health workforce, known as promotoras/es, whose main role is to provide outreach and education about chronic medical and mental health problems to the community.

### Participants

A total of 66 youth identifying as Latinx, between the ages of 8–17 (33.3% female; mean age = 11.74 years, SD = 2.86 years; 92.4% Latinx, 7.6% Mixed Latinx), completed the baseline measures that were used for this study. Inclusion criteria for the parent RCT included an established cut-off of greater than 25 on the Screen for Child Anxiety Related Emotional Disorders (SCARED) on either child or parent report (Birmaher, 2005). This cutoff has been found to discriminate anxious from non-anxious youth (Birmaher et al., 1999). Additionally, a measure of impairment, the Child Anxiety Life Interference Scale (CALIS-P; Orgilés et al., 2020), was used to ensure that there was some functional impairment associated with the anxiety symptoms. Child or parent participants had to endorse at least a 2 or greater on this scale to be eligible for the study. Parents and youth had to speak English or Spanish and be able and willing to provide informed parental consent/child assent. Participants with significantly elevated level(s) on other emotional and behavioral problems, such as depression, attention/hyperactivity, substance use, or disruptive behavior problems, that was considered clinically significant and primary, were not eligible and instead were referred for more appropriate services. Youth with significant medical and/or psychiatric conditions contraindicating study participation (e.g., suicidality, psychotic symptoms, mania, or autism spectrum disorders) were also excluded from the study. Additionally, youth who were in concurrent psychosocial treatment for anxiety were not eligible. Among youth using psychotropic medication, dosage had to be stable for at least 6 weeks prior to the study start date.

### Procedure

This study uses a cross-sectional, correlational design and only data from the baseline time point of the parent RCT are used for this study. All participants provided informed consent to participate in the study and received monetary compensation for their time. Full Institutional Review Board (IRB) approval was granted for this study. Parent–child dyads who consented to participate in the study provided demographic information, completed questionnaires, and participated in the clinical assessment of symptom severity in either English or Spanish, based on language preference. Performance-based measures of attention were administered to youth at baseline at the participant’s home by the promotoras/es. Promotoras/es also administered the self-report assessments to both parent and child at this visit. Clinician-administered assessments of symptom severity with parents and children (i.e., Pediatric Anxiety Rating Scale) were conducted by bilingual trained graduate-level assessors via phone. The baseline assessment took between one to two hours and is the only relevant timepoint for the current study.

#### Training

##### Promotores/As

The promotores/as were responsible for administering consents, questionnaires, and performance-based measures of attention, as well as other tasks related to the parent RCT. Pertinent to the current study responsibilities, the promotores/as engaged in self-guided didactics in which they read the IRB proposal and became familiar with all consenting and HIPAA procedures. They were also assigned readings on child anxiety disorders as well as attention bias assessment and training. Next, the promotores/as attended an in-person workshop, conducted by the Principal Investigators (PIs) of the study, which included presentations about study methods and procedures, role-plays of informed consent procedures, demonstration of the performance-based measures, mock role play of visits (including the baseline visit), and training in suicide risk assessment and crisis management. The promotores/as administered all procedures to at least two participants under direct supervision from the PIs and two additional cases were rated for fidelity. The promotores/as were expected to meet 80% agreement on the administration of the core components of the protocol (including administration of the assessments). Weekly supervision was provided by the PIs.

##### Assessors

The clinical assessment instrument consisted of a clinician-administered Pediatric Anxiety Disorders Ratings Scale (PARS) interview of anxiety symptomatology. The interviews were conducted by clinical psychology graduate students. Graduate student assessors were trained and supervised by a Ph.D. level psychologist, and reliability training consisted of didactic training, re-rating of previously recorded assessment tapes, and administration of assessments with live supervision from an experienced assessor.

#### Baseline Measures

##### Demographic Questionnaire

Parents completed a demographics information sheet that assessed youth age, youth and parental ethnicity/race, youth gender, highest parental education level, family income level, and parental employment status/occupation.

##### Anxiety. *Pediatric Anxiety Rating Scale* (Walkup et al., 2002)

The PARS is a clinician-rated measure incorporating parent and child report of anxiety symptoms and severity. The PARS consists of a 50-item anxiety symptom checklist and seven global severity/impairment items. Each item is scored using a 6-point scale (0 for none, and 1–5 for minimal to extreme), with a score of 3 on each item indicating a clinically significant level of severity or impairment. The PARS was translated into Spanish for this study using a forward and backtranslation methods, augmented by consensus meetings with bilingual clinicians with expertise in child anxiety. In line with previous landmark studies (Walkup et al., 2008), a total PARS score was calculated by summing items 2–7, with higher scores indicating higher levels of severity or impairment (Verhulst & van der Ende, 2006; Walkup et al., 2002, 2008). Consistent with previous research, item 1 was excluded in the total score calculation to minimize potential overestimation of total anxiety severity, as it asks for total number of anxiety symptoms (Walkup et al., 2008). The PARS has acceptable psychometric properties, with high inter-rater reliability (ICC = 0.97) and fair test–retest reliability (ICC = 0.55) (Walkup et al., 2002). Cronbach’s alpha for internal consistency ranges from 0.64 in clinical populations (Walkup et al., 2002) to 0.91 in nonclinical populations (Ginsburg et al., 2011). The PARS was completed at baseline and is the primary outcome measure in this study. In this study, the Cronbach’s alpha for the PARS for our total sample (n = 66) was 0.847. The Cronbach’s alpha was 0.857 and 0.866 for the English and Spanish versions of the PARS, respectively.

##### Attention Bias. *Attention Bias Assessment Task* (ABA Task)

The ABA Task is a modified version of the dot-probe paradigm similar to the original task used by MacLeod et al. (1986). Each trial begins with a fixation cross presented in the center of the computer screen for 500 ms. The cross is then replaced by a pair of faces, one angry and one neutral in expression, that are presented in a top–bottom manner in the center of the screen for 500 ms. Face stimuli are drawn from the NIMH Child Emotional Faces Picture Set (Egger et al., 2011). The face pair disappears and a probe (a cartoon bear holding a box in one hand) appears in the location of one of the two faces. The initial probe disappears and is replaced immediately at the same location by another bear holding two boxes, one of which is the same as the previous box. Participants are instructed to select the box that matches the previous box as quickly as possible. The probe remains on the screen until the participant responds. Response latencies to identify the probe is recorded from the onset of the presentation of the probe to the button press. A total of 256 trials is delivered during the attention bias assessment with the probe replacing the angry faces on 50% of the trials and the neutral face in the remaining half. Consistent with previous research (Mathews & MacLeod, 2005), we calculated an attentional bias index as the difference in mean response latencies between trials in which the probe replaced the neutral stimuli and trials in which the probe replaced the threat stimuli. Positive values reflect bias towards negative relative to neutral stimuli, whereas negative values reflect bias away from negative stimuli.

Informed consent was obtained from all individual participants included in the study. All authors certify that they have no affiliations with or involvement in any organization or entity with any financial interest or non-financial interest in the subject matter or materials discussed in this manuscript. Approval for this study was obtained from the Institutional Review Board of the University of Los Angeles, California. The procedures used in this study adhere to the tenets of the Declaration of Helsinki.

### Data Analytic Plan

**Fig. 1:**
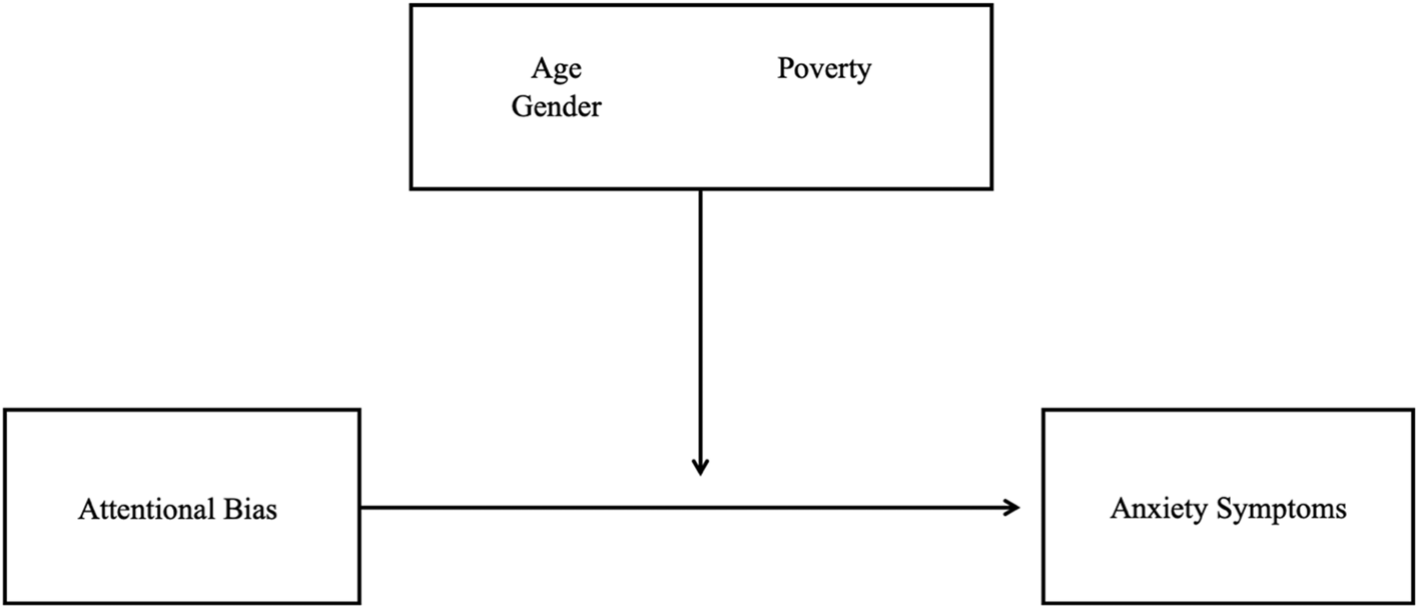
Moderation model of sociodemographic factors on relationship between attention bias and anxiety symptoms

Cross-sectional analyses were conducted using the baseline data from the RCT. Moderation analyses were conducted using the PROCESS macro for SPSS (Hayes, 2017), a regression-based methodology with bootstrapping. The models included baseline attention bias as the independent variable, the clinician-rated PARS measure for anxiety as the dependent variable, and sociodemographic variables as the moderators (see Fig. 1). Each sociodemographic variable (i.e., age, gender, and poverty) was included as a moderator separate models and therefore, three total regression models were run. Consistent with Reinholdt-Dunne et al. (2012), we divided youths into two age groups. Specifically, youths ages 11 years and older were classified as older youth (n = 36) and youths ages 10 and younger were classified as younger youth (n = 30). We therefore used another dummy code for whether children were older youth (1) or younger youth (0).

Based on the federal cutoffs for living below the poverty line in California (Department of Health & Human Services, 2019), we implemented a dummy code for whether families were living in poverty considering their household size (1) or if they were not living in poverty considering their household size (0). All models employed 10,000 bootstraps and variables that defined products were mean-centered. An Analysis of Variance (ANOVA) test was performed to examine differences between the income groups on severity of anxiety. Similarly, to ensure youth below and above the poverty line were comparable on gender and age (younger or older youth), chi-square tests of independence were conducted.

Consistent with previous research (Pine et al., 2005; Roy et al., 2008), participants who had accuracy levels less than 75% in the ABA Task were excluded from analyses (n = 4). Trials below 200 ms and above 2500 ms were also excluded, per past research indicating that latencies outside of this range could be due to a lapse of focus on the task, computer difficulties with button registration, or higher-order processing that may be too controlled for inclusion within a performance-based interpretation task (Rozenman et al., 2011, 2014). Next, consistent with previous research (MacLeod & Mathews, 1988), we calculated bias scores by subtracting each participant’s mean response latency on threat-congruent trials (i.e., probe appeared in the prior location of the threatening face) from their mean response latency on threat-incongruent trials (i.e., probe appeared in the location of the neutral face). Thus, larger bias scores indicate faster responding to the threat congruent trials and hence greater attentional bias toward threat-related information. All analyses were conducted with an alpha level of 0.05, and no corrections were made for multiple comparisons. We estimated individual linear regression models with attention bias score, each sociodemographic variable (i.e., age, gender, poverty level), and their interaction.

## Results

**Fig. 2:**
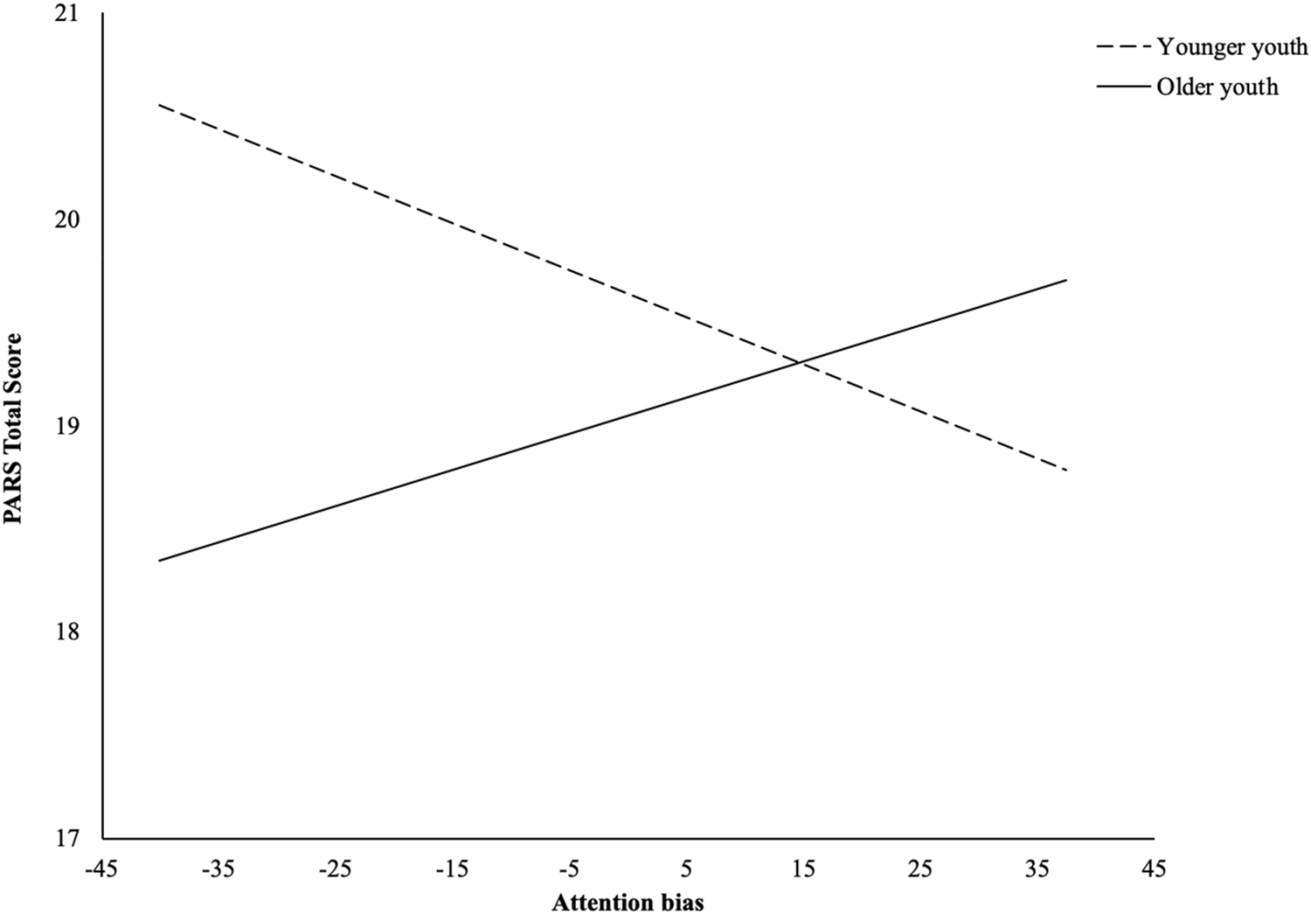
Moderation model of age on relationship between attention bias and anxiety symptoms

Table 1 shows baseline demographic information and Table 2 summarizes the interaction terms for the moderating effect of age, gender, and poverty between attention bias and anxiety scores. The first model testing whether the association between attention bias and anxiety was moderated by age was not statistically significant (*R*^2^ = 0.043, *F* (3, 62) = 0.938, *p* = 0.428). The association between attention bias and anxiety was not statistically significant (*β* = − 0.781, *t* = − 1.609, *p* = 0.113). The association between age (i.e., older youth and younger youth) and anxiety was not statistically significant (*β* = 0.066, *t* = 0.356, *p* = 0.723). The interaction between attention bias and age was not statistically significant (*β* = 0.006, *t* = 1.673, *p* = 0.099; see Fig. 2).

**Table 1:** Baseline characteristics of sample

Characteristic	n = 66
Age (mean years, SD)	11.74, 2.86
Sex (male n, %; female n, %)	44, 66.7%; 22, 33.3%
Race/Ethnicity (Latinx n, %; Mixed Latinx n, %)	61, 92.4%; 5, 7.6%
Child country of birth (US-born %, outside US %)	86.4%, 12.1%
Child’s grade in school (mean grade, SD)	6.38, 2.82
Living above poverty, considering household size	27, 40.9%
Living in poverty, considering household size	39, 59.1%
English-speaking parents; Spanish-speaking parents (n, %)	23, 34.8%; 43, 65.2%
English-speaking youth; Spanish-speaking youth (n, %)	55, 83.3%; 11, 16.7%

**Table 2:** PARS × attention bias moderation models for poverty, gender, and age

Total sample	N = 66
Mean PARS	19.03
Mean attention bias score	0.685

Moderation models				
		t	*df*	*p*
*Poverty*				
Youth living in poverty (n = 39)				
Mean PARS	19.59			
Mean attention bias score	− 2.88			
Youth living above poverty (n = 27)				
Mean PARS	18.37			
Mean attention bias score	3.03	− 3.17	1062	0.002***
*Age*				
Younger children (n = 30)				
Mean PARS	19.37			
Mean attention bias score	11.90			
Older children (n = 36)				
Mean PARS	18.86			
Mean attention bias score	− 8.91	1.67	1.62	0.099
*Gender*				
Female (n = 22)				
Mean PARS	19.52			
Mean attention bias score	8.45			
Male (n = 44)				
Mean PARS	18.80			
Attention bias score	− 2.31	0.324	1.62	0.747

**Fig. 3:**
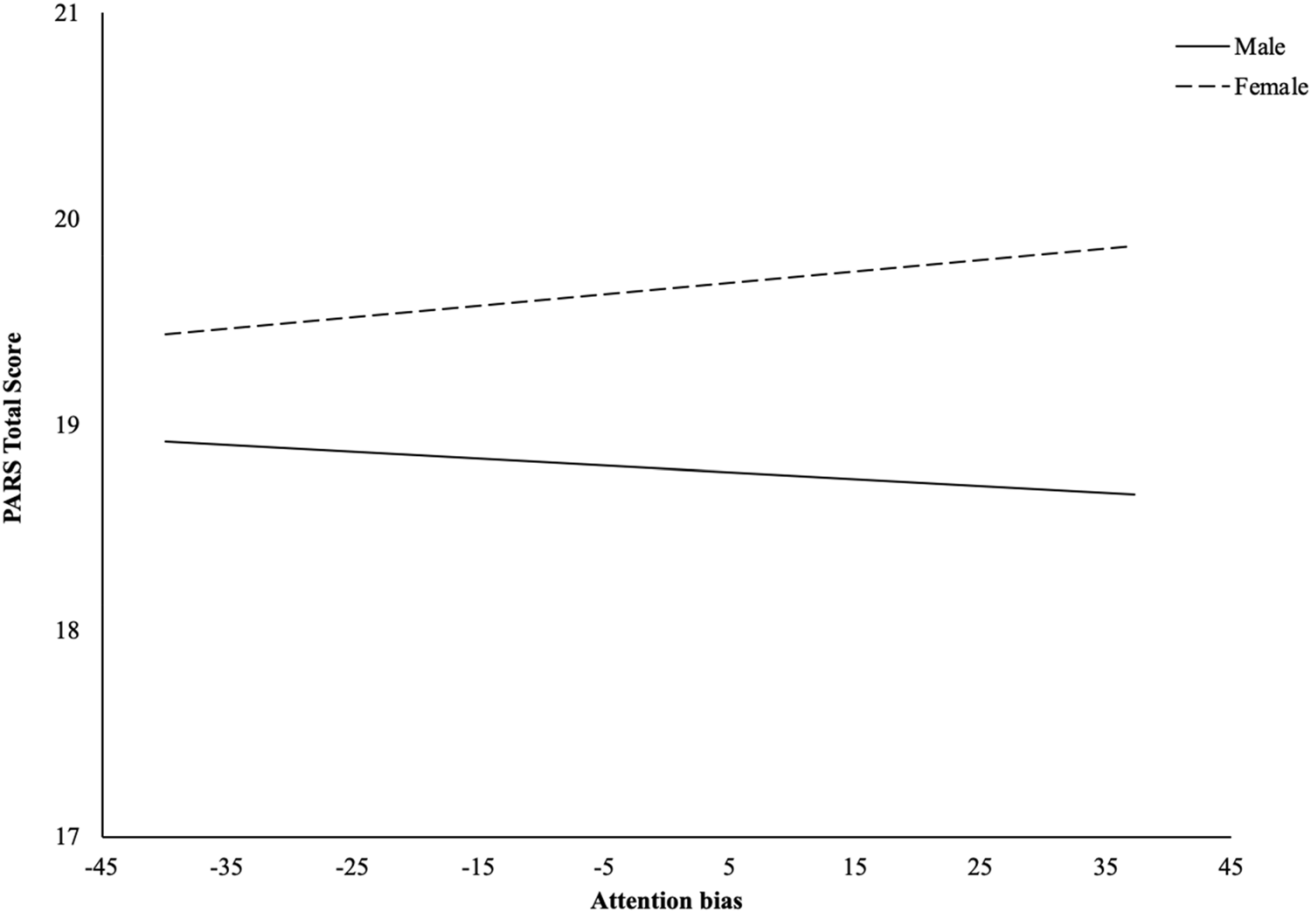
Moderation model of gender on relationship between attention bias and anxiety symptoms

The second model testing whether the association between attention bias and anxiety was moderated by gender was not statistically significant (*R*^2^ = 0.017, *F* (3, 62) = 0.265, *p* = 0.851). The association between attention bias and anxiety was not statistically significant (*β* = − 0.003, *t* = − 0.232, *p* = 0.818). The association between gender (i.e., male and female) and anxiety was not statistically significant (*β* = 0.876, *t* = 0.815, *p* = 0.418). The interaction between attention bias and gender was not statistically significant (*β* = 0.009, *t* = 0.324, *p* = 0.747; see Fig. 3).

**Fig. 4:**
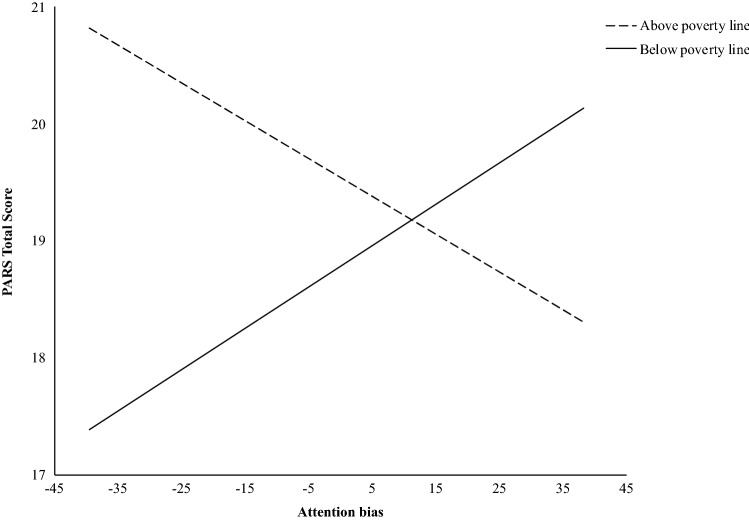
Moderation model of poverty on relationship between attention bias and anxiety symptoms

The third model testing whether the association between attention bias and anxiety was moderated by poverty was statistically significant (*R*^2^ = 0.136, *F* (3, 62) = 2.93, *p* < 0.05). The association of attention bias and anxiety was statistically significant (*β* = 0.072, *t* = 2.847, *p* = 0.006). The association of poverty (i.e., not living in poverty and living in poverty) and anxiety was not statistically significant (*β* = 1.385, *t* = 1.455, *p* = 0.151). The interaction between attention bias and poverty was statistically significant (*β* = − 0.090, *t* = − 3.165, *p* = 0.002). Probing of the interaction showed significant effects of attention bias at the poverty level (*θ*_X→Y|W=0_ = 0.072, *p* < 0.01), though insufficiently significant effects of attention bias above the poverty level (*θ*_X→Y|W=1_ = − 0.018, *p* = 0.171). Specifically, youth living in poverty had lower attention bias (i.e., a decreased vigilance response to negative faces) than youth who were not living in poverty, which was associated with increased levels of anxiety (see Fig. 4).

The one-way ANOVA assessing whether there was a statistically significant difference in anxiety levels between youth above or below the poverty line was not significant (*F* (1.64) = 1.469 *p* = 0.230). Additionally, the chi-square tests examining differences in youth’s gender and age (younger vs. older youth) across poverty level were not significant, (*χ*^2^(1) = 2.538, *p* = 0.111) and (*χ*^2^(1) = 1.306,* p* = 0*.*253), respectively.

## Discussion

The present study sought to address a gap in the literature by testing plausible demographic and socioeconomic variables as moderators of the association between attention bias and anxiety. Given previous findings supporting the role of sociodemographic characteristics, we examined the effect of age, gender, and poverty on attentional bias toward threat in a sample of underserved, Latinx youth with clinical levels of anxiety. The current study improved our understanding of anxiety among rural, Latinx youth, a population that has been largely ignored in the research literature. Specifically, moderation analyses identified for whom cognitive processes (i.e., attention bias) associated with anxiety may be most impactful.

Consistent with the extant literature linking socioeconomic status with child well-being (Bradley & Corwyn, 2002; Loomis, 2021), results revealed that level of poverty affected the relationship between attention bias and anxiety. However, contrary to previous research (Dufford et al., 2019; Raver et al., 2017), youth living below the poverty line displayed an attention bias *away* from threat in comparison to youth living above the poverty line, who displayed an attention bias *towards* threat. Among youth living below the poverty line, attention bias away from threat was associated with increased levels of anxiety, a novel finding in the literature.

Youth living in households struggling with chronic poverty may be at increased risk of exposure to adversity, such as greater levels of community and family violence, and resource instability. It has been proposed that high exposure to adversity could result in youth developing an attention bias away from threat as a protective mechanism, where attentional avoidance serves to minimize the distressing mood state elicited by the aversive stimuli (Mogg & Bradley, 1998). Laboratory-based research with adults has found that acute stress can lead anxious individuals to shift their attention away from threat (Amir et al., 1996; Garner et al., 2006; Helfinstein et al., 2008; Mansell et al., 1999; Mathews & Sebastian, 1993). Similarly, in at risk samples, adults who have experienced extreme trauma, such as child abuse or combat, exhibit an attention bias away from threat in studies that use variants of the dot-probe task (Bar‐Haim, 2010). These findings also extend to youth who have been physically abused, as well as those who meet criteria for posttraumatic stress disorder (Pine et al., 2005). Given these findings, it may be that trauma, specifically traumas of an interpersonal nature, lead to a bias *away* from threat due to the implicit tendency to avoid social cues. However, in this study, bias away from threat was associated with heightened levels, rather than decreased levels of anxiety. Some have proposed that avoidance of detailed processing of threatening cues, such as social cues (i.e., disengaging attention away from threatening faces), replaced by facilitated attention to non-threatening cues (i.e., the neutral probes), may maintain anxiety among certain groups of individuals (Vassilopoulos, 2005), leading to higher levels of anxiety in those who have a tendency to use attentional avoidance.

Interestingly, youth in the current study are from a rural Latinx community, where there are high rates of being exposed to at least one traumatic event before adulthood (De Silva et al., 2020), which may be associated with differential information processing of threat cues. Risk associated with an increased exposure to traumatic experiences, including traumas of an interpersonal nature, may be further exacerbated by chronic poverty which is prevalent in many rural communities (Crouch et al., 2000; López et al., 2017). There is a dearth of studies that examine poverty-related adversity and its effects on attention bias and anxiety—our study underscores a need to examine the impact of these experiences more closely. In addition to examining economic disadvantage based on income, there is a need to further examine other social determinants of mental health (e.g., maltreatment, food insecurity, neighborhood context) in order to fully understand how poverty impacts the relationship between attention bias and anxiety.

Despite data suggesting there are shifts in attention bias across age and development, with older youth showing a preference for negative stimuli (Burris et al., 2017; Elam et al., 2010; Gross et al., 2006), age did not moderate the relationship between attention bias and anxiety in this sample. Though a previous study found age to be a significant moderator of the association between attention bias and anxiety (Carmona et al., 2015), findings from this study may have been influence by a small sample size (n = 33) and the use of word stimuli instead of face stimuli—these findings have not been replicated with larger samples. Indeed, attention bias paradigms administered in research studies are variable and often do not take into account developmental issues that could be of concern, such as cognitive skills and capacities that may be required for effective task completion (Abend et al., 2019). Tasks measuring attention bias require rapid engagement, as well as psychomotor and cognitive learning, and engagement within these tasks has been a limitation of studies using standard attention bias paradigms (Carmona et al., 2015). These capacities mature and improve throughout childhood, reaching an optimal functionality in adolescence and early adulthood. Future studies that include larger sample sizes and consider the developmental appropriateness of attention bias tasks are necessary.

In our sample, no differences in attention bias or anxiety were found between girls and boys. These findings are inconsistent with studies that have found gender differences in youth negative interpretation bias, a cognitive process that is related but distinct from attention bias. For example, Gluck et al. (2014) found that adolescent girls were more likely to note negative interpretations than same-aged boys when presented with ambiguous scenarios. Similarly, Salemink and Wiers (2011) found that adolescent girls were more anxious and had a stronger tendency to interpret ambiguity negatively relative to adolescent boys. The lack of study findings might be related to the differences between paradigms assessing negative interpretation bias versus attention bias. Nonsignificant findings may also be influenced by the disproportionate number of boys versus girls (n = 22 females; n = 44 males) in this study, which may limit the conclusions that can be made about gender as a moderator in this study.

In general, methodological issues may explain inconsistent findings of the current study, as well as the larger attention bias and anxiety literature. For example, there is wide variability in the type of threatening stimuli used in the dot-probe tasks (Zvielli et al., 2014), and it is unclear if different kinds of threatening stimuli are perceived similarly across cultures. Ethnicity and race-related differences in threat-related perceptual decision-making have been found in recent studies—these are theorized to stem from top-down factors (i.e., exposure to, previous experience with, and expectations associated with the race of stimuli faces) that influence threat identification and subsequent decisions (Glasgow et al., 2020). For example, in a specific population such as Latinx youth with income-related concerns, angry faces of White non-Latinx males may not be inherently threatening. Specific learning and unique contextual factors, such as increased risk of experiencing a traumatic event or poverty may exert an important influence on attention bias towards threat (Zvielli et al., 2014). The phenomenon of whether individuals attend or disengage from threat, and to which threatening stimuli they attend to, has been shown to be a function of motivational relevance of threatening stimuli and past learning specific to certain forms of threat. The variability of whether youth attend to or disengage from threat in attention bias paradigms could be due to a lack of nuanced consideration of sociodemographicl variables, such as whether youth are living in poverty. Future studies should pay greater attention to the cultural salience of stimuli that are being used in these paradigms.

Additionally, longitudinal studies are needed to understand the causal influence of attention biases on anxiety and how economic hardship may interact with such biases to impact mental health outcomes. Of note, attention bias *toward* versus *away* from threat is relative to other participants in the sample, and research remains unclear on how far away a bias score should be from zero in order to indicate bias toward or away from threat. Further, research on attention bias has not yet established how much bias in either direction is beneficial or disadvantageous to individuals (Beard, 2011). Future investigations should examine how much bias in either direction leads to significant psychological sequelae. Additionally, future studies should examine adversity beyond economic status, including exposure to violence, maltreatment, and food insecurity in order to better assess specific aspects of adversity that impact this relationship. Future studies investigating these factors should include non-anxious youths to determine whether poverty exerts a differential effect on the relationship between attention bias and anxiety among clinically anxious when compared to non-anxious youth. Lastly, our study focused on an understudied and difficult-to-recruit sample, and therefore had a small sample size (n = 66). Given the exploratory nature of our study, we did not conduct an a-priori power analysis. Though our sample size is larger than other studies with hard-to-reach communities, the robustness of our findings is limited by sample size considerations.

Despite these limitations, our findings make a case for examining economic disadvantage when trying to understand the relationship between attention bias and youth anxiety. To our knowledge, this is the first study to examine potential moderators of the relationship between attention bias and anxiety in a sample of rural Latinx youth. The results of this study suggest that poverty affects risk for anxiety through cognitive processes and raises questions regarding the direction of this association for minoritized groups. Our study brings attention to the consideration of varying pathways by which poverty may exert its influence, such as previous trauma exposure, chronic stress and financial instability. In fact, previous models have posited that chronic stress related to poverty can affect biological regulatory systems, interacting with genetic polymorphisms to increase hypervigilance to the environment (Brody et al., 2013; Evans & Kim, 2013). Pending replication with a larger sample size, this could suggest that interventions for youth with anxiety who are living under the poverty line may need to consider whether there are unique cognitive risk factors specific to this group (including possible trauma and chronic stress) and whether treatment strategies need to be tailored accordingly.

## Summary

Attention bias has been proposed as a variable that confers risk for anxiety development in children, however, the influence of sociodemographic variables on the relationship between attention bias and anxiety remains unclear. This study examined the relationship between attention bias and anxiety in a sample of rural Latinx youth and investigated potential sociodemographic moderators of this relationship. Results demonstrated no moderating effects for age or gender. Novel findings revealed that, contrary to prior research, youth below the poverty line displayed an attention bias *away* from threat in comparison to youth who were above the poverty line, who displayed an attention bias *towards* threat. Among youth who were below the poverty line, attention bias away from threat was associated with increased levels of anxiety. Pending replication, these findings have potential implications for clinical practice, and highlight the importance of economic adversity in understanding the relationship between attention bias and anxiety.
